# Supplementary material for: Rapid and High Throughput Hydroponics Phenotyping Method for Evaluating Chickpea Resistance to Phytophthora Root Rot
Source: Plants (Basel). 2023 Dec 4;12(23):4069. doi: 10.3390/plants12234069 (PMC10708383; doi:10.3390/plants12234069)
Supplement: Supplementary file 1 [file plants-12-04069-s001.zip › plants-2722115-supplementary.pdf]

Supplementary Materials

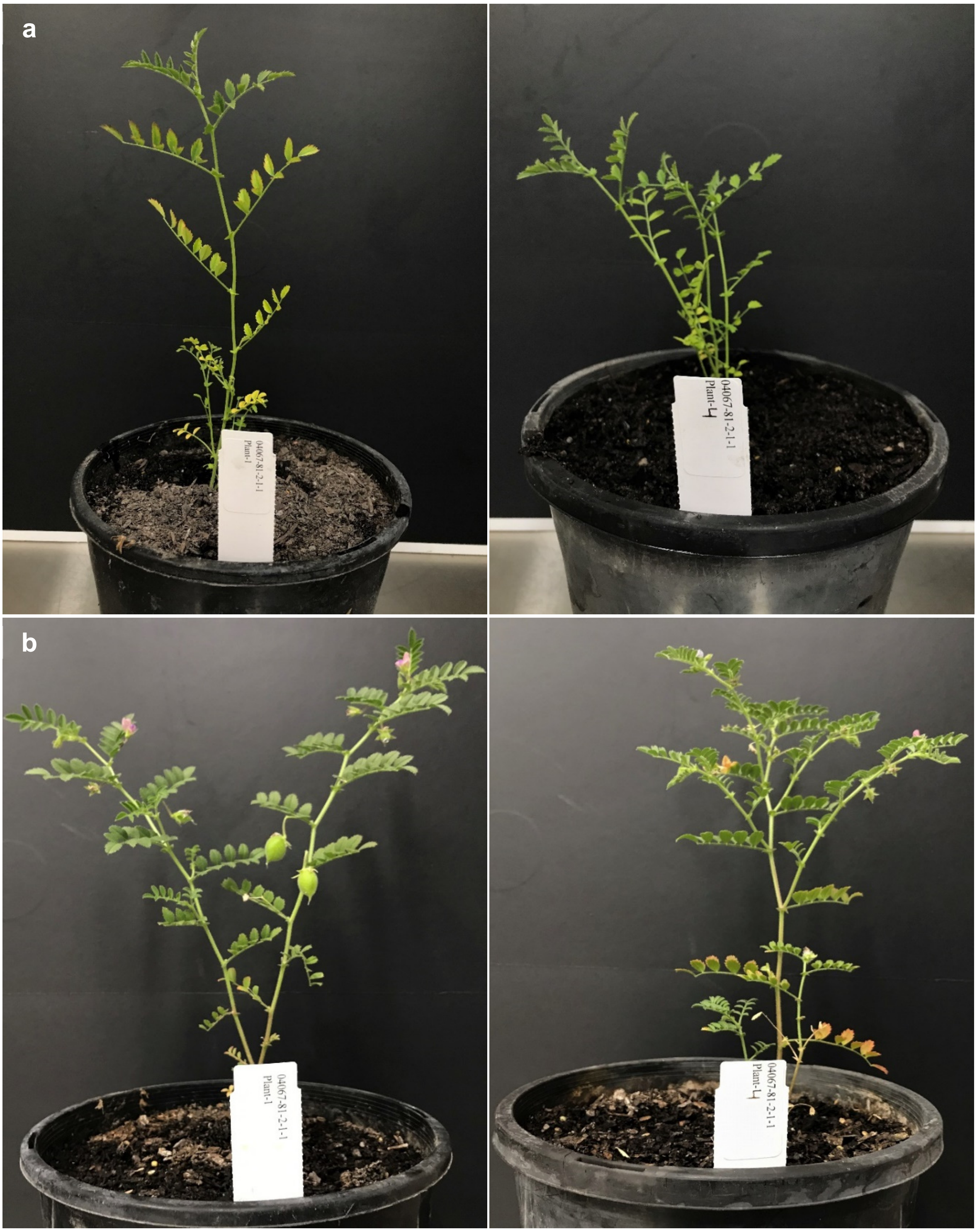

Supplementary Figure S1. Phenotypic differences between (a) plants at the time of transplanting to potting mix post-phenotyping E1 and (b) 5 weeks later showing growth and pod development.

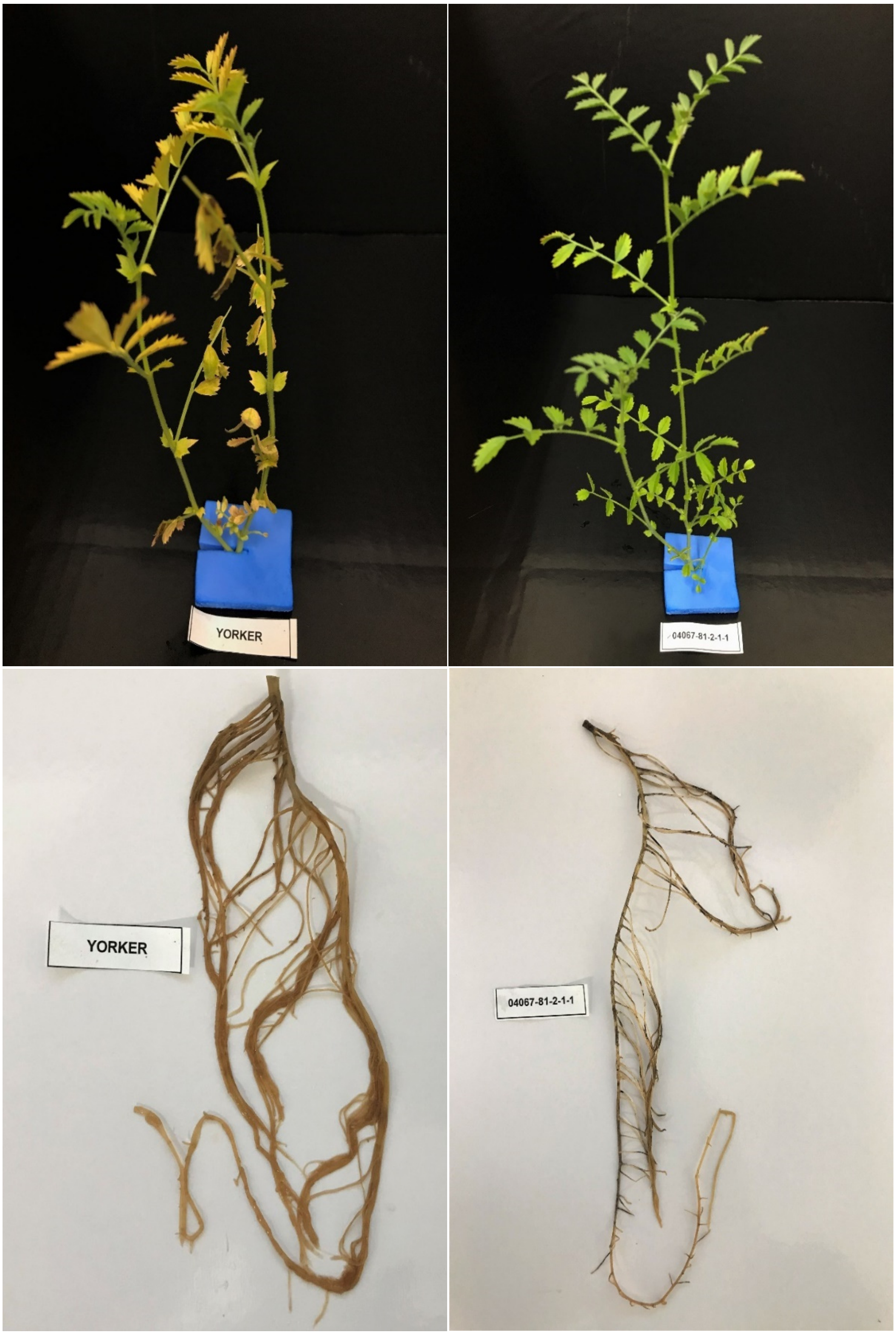

Supplementary Figure S2. Phenotypic difference between shoot and roots of genotypes Yorker (left) and 04067-81-2-1-1 (right) 11 days after inoculation with *P. medicaginis* in E2.

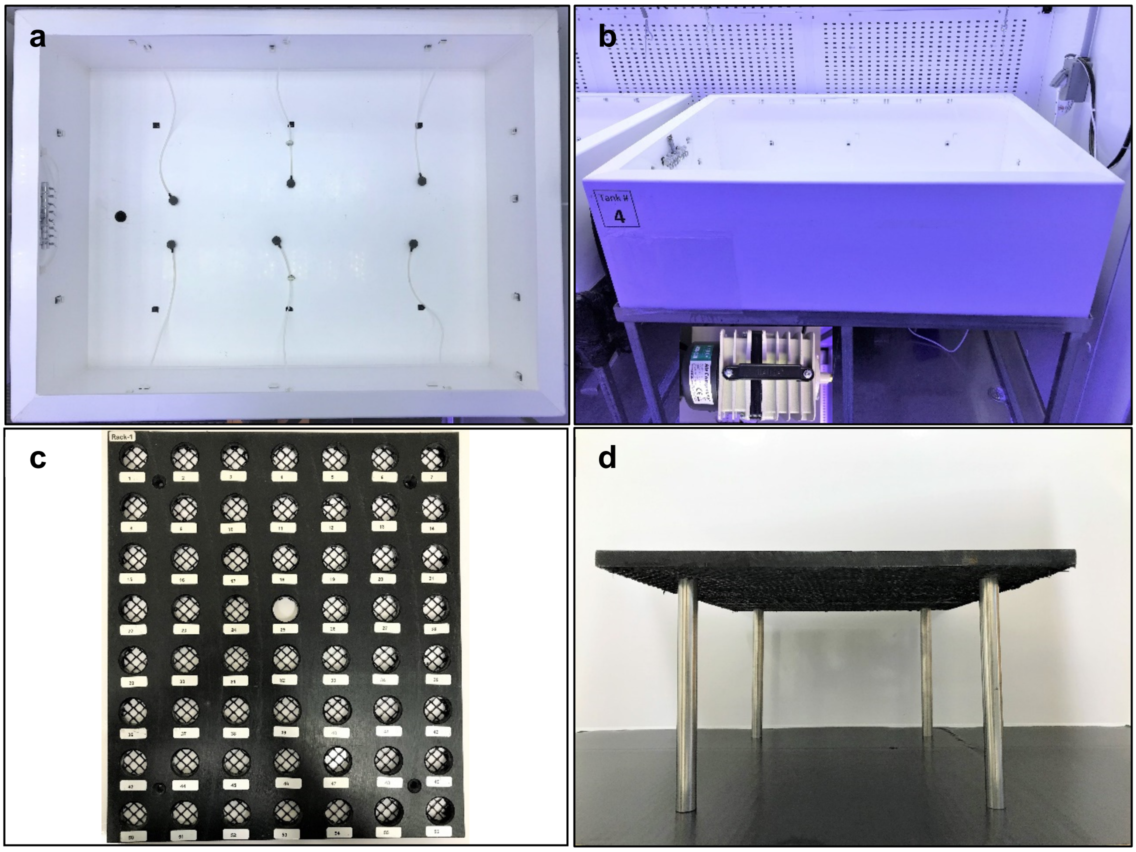

Supplementary Figure S3. Hydroponics setup used for PRR screening experiments: (a) a view of inside the tank showing six air stones connected with air pump; (b) side view of tank and air pump; (c) top view of individual rack used for growing plants; (d) side view of rack with legs attached.

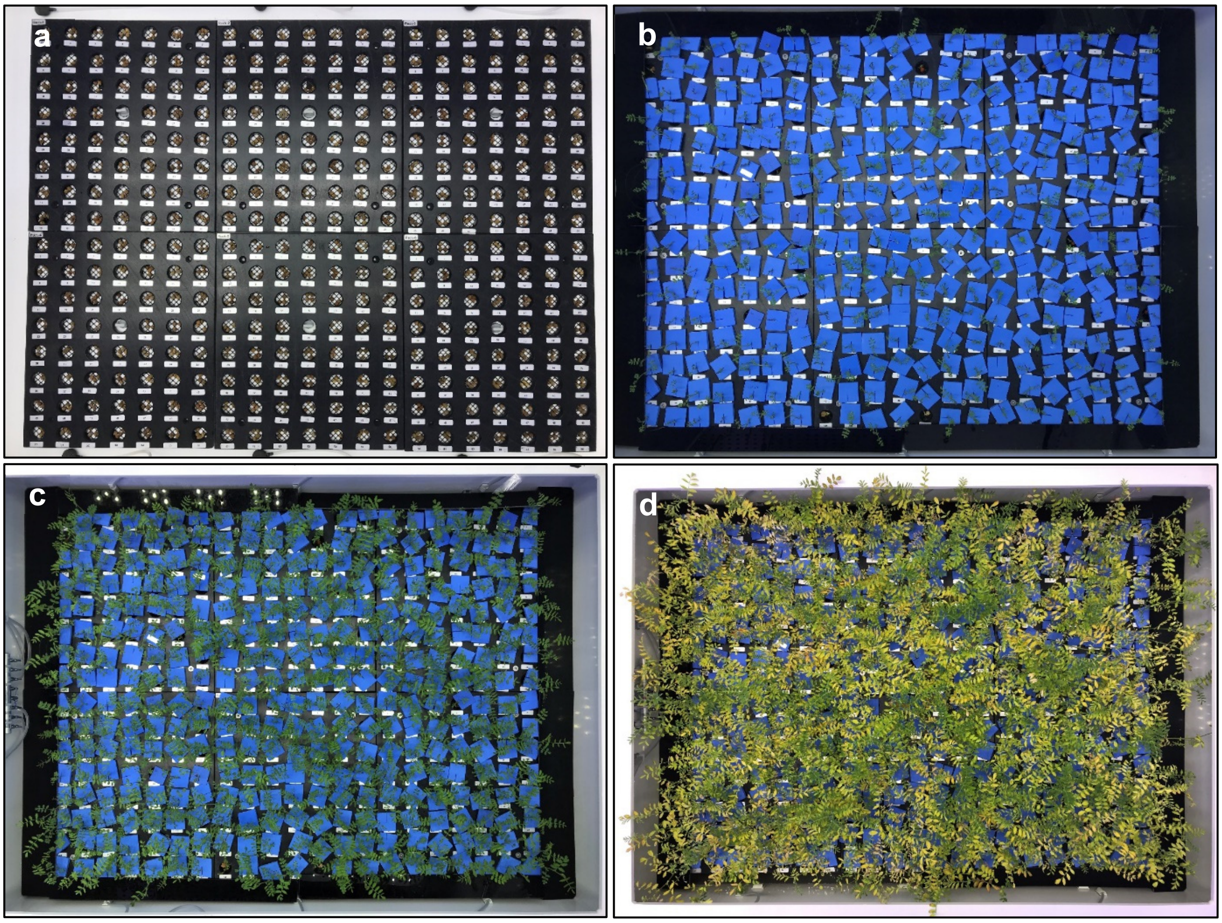

Supplementary Figure S4. Different growth stages of PRR disease screening hydroponics experiment: (a) top view of six racks with seeds inside a tank; (b) seedlings were thinned to single plant per hole; (c) plant phenotype before inoculation; (d) plant phenotype at 12 days after *P. medicaginis* inoculation.

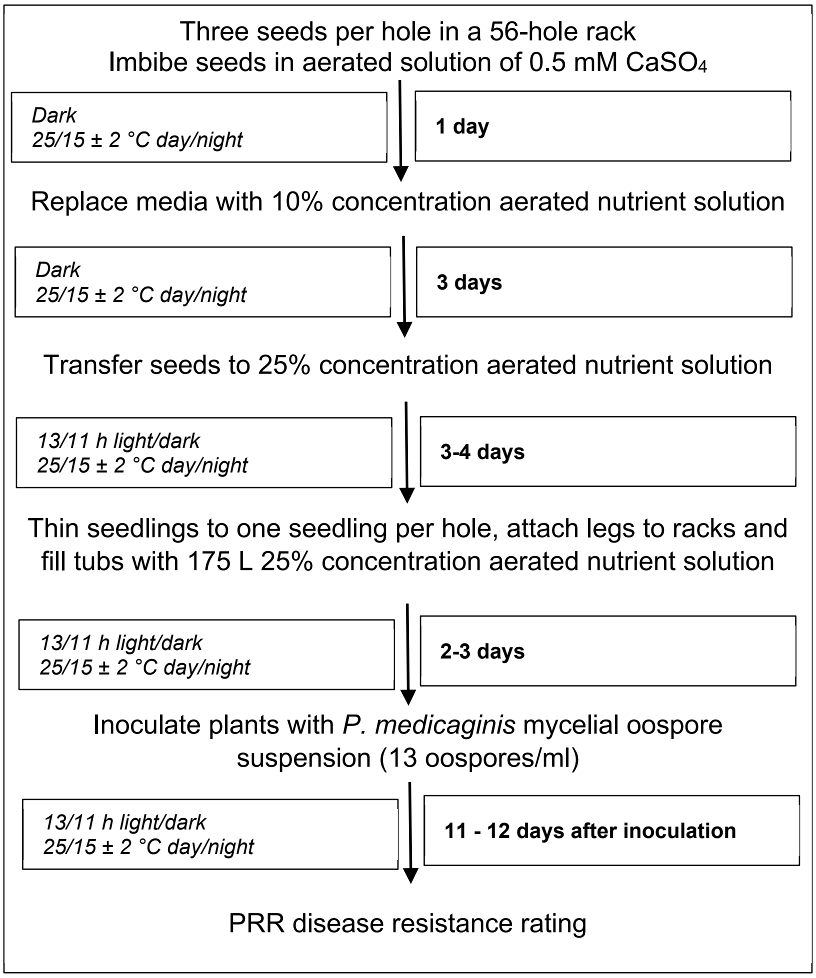

Supplementary Figure S5. Flow chart outlining the timeline and key steps involved in the rapid hydroponic process. Temperature and photoperiod are given on left of arrows, whereas the timing on right of arrows indicate time between steps.
